# The cGAS-STING Pathway in Pulmonary Diseases: Mechanisms and Therapeutic Potential

**DOI:** 10.3390/ijms262110423

**Published:** 2025-10-27

**Authors:** Zhuo Zhang, Jiacheng Jiang, Guodong Wu, Xueping Wei, Yakun Weng, Long Shuang Huang

**Affiliations:** 1 Shanghai Frontiers Science Center of Drug Target Identification and Delivery, School of Pharmaceutical Sciences, Shanghai Jiao Tong University, Shanghai 200240, China; zzhuo1220@sjtu.edu.cn (Z.Z.); cc0320@sjtu.edu.cn (J.J.); guodongwu@sjtu.edu.cn (G.W.); happyforever@sjtu.edu.cn (X.W.); wykkkk0722@sjtu.edu.cn (Y.W.); 2 National Key Laboratory of Innovative Immunotherapy, Shanghai Jiao Tong University, Shanghai 200240, China

**Keywords:** cGAS-STING, pulmonary disease, STING inhibitor

## Abstract

The cyclic GMP-AMP synthase–stimulator of interferon genes (cGAS-STING) pathway, a central innate immune sensor of cytosolic DNA, plays a dual role in immunoregulation within pulmonary diseases. Recent studies demonstrate its critical role in sensing microbial infections and tissue injury in the lung, allowing it to drive the production of type I interferons (IFN-I) and pro-inflammatory cytokines. While this pathway is essential for anti-viral defense and anti-tumor immunity, its dysregulation can exacerbate pathologies such as chronic obstructive pulmonary disease (COPD), pulmonary fibrosis, and lung cancer, mainly through sustained inflammation and fibroblast proliferation. Nowadays, many cGAS-STING agonists and inhibitors are available to treat different diseases. This review comprehensively summarizes the basic mechanism of the cGAS-STING pathway, its diverse roles across various pulmonary diseases, and the current landscape of potential therapeutic strategies targeting this pathway. Notably, the critical role of the cGAS-STING signaling pathway in various lung diseases offers new avenues for therapeutic research.

## 1. Introduction

**Figure 1:**
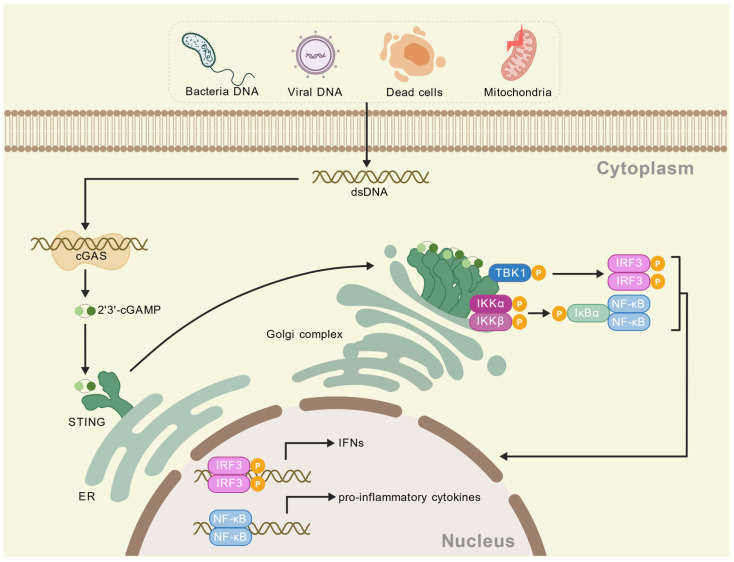
The mechanism of the cGAS-STING pathway. When pathogens such as bacteria and viruses invade the body, they release dsDNA; this also includes DNA derived from mitochondria dysfunction and dead cells. These dsDNA molecules are recognized and bound by cGAS, which then catalyzes the production of cGAMP. cGAMP can bind to and activate STING. Upon activation, STING undergoes conformational changes and dimerization and then translocates from the endoplasmic reticulum (ER) to the perinuclear region via the Golgi apparatus. Activated STING serves as a scaffold to recruit and activate the kinase TBK1. Subsequently, TBK1 phosphorylates the transcription factor IRF3. Phosphorylated IRF3 forms a dimer, translocates to the nucleus, and cooperates with other factors (e.g., NF-κB) to initiate the transcriptional expression of IFN-I and various inflammatory cytokines.

The innate immune system is a frontline defense against microbial invasion. Among its components, pattern recognition receptors (PRRs), including the Toll-like receptors (TLRs) family and Toll/interleukin-1 receptor (TIR) family [1], represent the most extensively studied class of cell membrane receptors. Upon recognition of pathogen-associated molecular patterns (PAMPs), PRRs are activated and subsequently initiate downstream inflammatory cascades, which may ultimately contribute to the development of various inflammatory pathologies. Cytoplasmic double-stranded DNA (dsDNA) sensor cyclic GMP-AMP synthase (cGAS) was initially identified as a key mediator in the induction of IFN-I and nuclear factor-kappa-B (NF-κB) p65 responses [2]. The cGAS-STING signaling pathway can recognize both exogenous and endogenous DNA. Upon DNA recognition, cGAS produces 2′ to 3′ cyclic GMP-AMP (cGAMP) as a second messenger, which binds to and activates the stimulator of interferon genes (STING, also known as TMEM173, ERIS, MPYS, and MITA) [2]. This process activates TANK-binding kinase 1 (TBK1), leading to the phosphorylation of interferon regulatory factor 3 (IRF3) and NF-κB, subsequently inducing IFN-I responses [3] (Figure 1).

The cGAS-STING signaling pathway has been recognized as a critical mediator in the pathogenesis of various diseases, particularly in pulmonary disorders. Numerous studies have aimed to elucidate its functional roles in the lungs and its therapeutic potential. This review systematically summarizes recent research advancements related to the cGAS-STING pathway in pulmonary diseases, with a specific focus on elucidating its multi-faceted roles and underlying molecular mechanisms across different respiratory conditions.

## 2. cGAS-STING Pathway

STING was first identified in 2008 [4], and subsequent research has confirmed its ability to induce IFN-I by activating IRF-3. IFN-I is a major anti-viral molecule in innate immunity [5]. Subsequently, Wu [6] et al. discovered that the induction of foreign DNA or DNA virus into mammalian cells triggers the production of cGAMP, which binds and activates STING. Activated STING undergoes a conformational change to facilitate its translocation from the ER to the Golgi [7], leading to IRF-3 activation and interferon-β (IFN-β) production.

However, the concrete mechanisms underlying these processes had remained unclear until Sun [8] et al. identified cGAS. The C-terminus of human cGAS (h-cGAS) can bind to the sugar-phosphate backbone of DNA in the cytoplasm and produce cGAMP to activate this pathway [9]. cGAMP contains two phosphodiester bonds: one is between GMP’s 2′OH and AMP’s 5′phosphate, and the other is between AMP’s 3′OH and GMP’s 5′phosphate [10]. Furthermore, the synthesis of cGAMP is considered a crucial step in this process. Although transient activation of STING can produce anti-viral or anti-inflammatory effects, persistent activation can lead to the development of inflammatory diseases.

In addition to producing IFN-I upon activation of this pathway, the NF-κB signaling pathway is also activated, leading to the production of pro-inflammatory cytokines (e.g., tumor necrosis factor [TNF], IL-6, and IL-1β) [11]. Apart from the canonical cGAS-STING pathway for IFN-I production, some noncanonical signals downstream of STING serve distinct functions. For example, the STING-PERK signaling pathway functions independently of the canonical pathway, suggesting that it is not related to the STING-TBK1-IRF3 pathway. The main function of this pathway is to modulate cellular senescence [12].

## 3. Activations and Functions

### 3.1. Conditions for cGAS-STING Pathway Activation

The DNA that activates cGAS is primarily derived from extracellular pathogens such as bacteria, viruses, or retroviruses, and damaged nuclei or mitochondria, which serve as self-DNA sources, constitute the primary sources [13]. Due to less efficient DNA repair capacity, mitochondrial DNA is more susceptible to damage then release compared to nuclear DNA [14]. Mitophagy, an important mechanism to clear damaged mitochondria, can reduce inflammation activation [15]. Furthermore, pathogens and cellular stress can generate different signals that affect mitochondrial integrity, and cells undergoing non-apoptotic death can release DNA. When this pathway is activated, it primarily exerts anti-viral effects, but also induces some pro-inflammatory effects depending on the duration of activation. In addition to these effects, danger sensing also plays a protective role through cGAS [16]. Importantly, these different types of activation are not mutually exclusive and can work synergistically in certain situations.

Apart from cGAS, a DNA sensor located in the cytoplasm, Z-DNA binding protein 1 (ZBP1) and absent in melanoma 2 (AIM2) also serve as sensors for cytosolic DNA. ZBP1, also known as DLM-1, contains Zα and Zβ domains, N-terminus, and a third DNA binding region [17], and especially binds to Z-conformation dsRNA. It was the first identified DNA sensor in the cytoplasm [18]. Upon binding to dsRNA, ZBP1 regulates innate immune activation and programmed cell death in inflammation-related diseases [19].

### 3.2. Biological Functions of cGAS-STING Pathway

The cGAS-STING pathway is a pivotal component in human health and disease processes. IFN-I has an anti-viral function, and mounting evidence indicates that the cGAS-STING pathway also contributes to anti-viral functions [20]. Furthermore, the cGAS-STING pathway induces IFN-I-mediated immunopathology in COVID-19 [21]. The activation of the cGAS-STING pathway in cells or tissues is specific, and the consequences of cGAS-STING pathway activation may vary across different types of lung cells, such as epithelial cells, endothelial cells, fibroblasts, macrophages, and neutrophils. In epithelial cells, particularly alveolar epithelial cells (AECs), the activation of the cGAS-STING pathway is considered the initiating and central mechanism in the pathogenesis of idiopathic pulmonary fibrosis (IPF). Sustained DNA damage signals drive the processes of chronic inflammation, cellular senescence, and fibrosis [22]. When STING is expressed in endothelial cells, exposure to cGAMP induces their activation and apoptosis [23]. Han [24] et al. demonstrated that following the development of airway allergic inflammation, the deletion of cGAS in AECs attenuates ovalbumin (OVA)- or house dust mite (HDM)-induced Th2 immune responses in mice.

Excessive activation of neutrophils is one of the pathological features of acute lung injury/acute respiratory distress syndrome (ALI/ARDS) [25]. Among the underlying mechanisms, neutrophil extracellular traps (NETs) impair pulmonary parenchymal cells and pulmonary immune cells, triggering an inflammatory cascade that exacerbates lung injury. Notably, NETs can be phagocytosed by macrophages, which in turn activates the cGAS-STING pathway, ultimately leading to the production of inflammatory cytokines [22].

#### 3.2.1. cGAS-STING Pathway and Cell Death

The cGAS-STING pathway is not exclusively associated with a particular mode of death but is directly or indirectly involved in multiple death pathways through IFN/ISG [7]. STING is related to antiproliferative cell states, which include cellular senescence and cell death [15]. Following the phosphorylation of IRF3, STING has been shown to interact with the pro-apoptotic proteins BAX and BAK, thereby triggering transcription-independent apoptosis activation downstream of STING [26,27]. The release of mtDNA into the cytosol can subsequently lead to the activation of the cGAS-STING pathway. In addition, when apoptosis is inhibited, STING-mediated activation of IFN-I and TNF signaling promotes necroptosis in a RIPK3-dependent manner [28]. STING induces membrane permeabilization through lysosomal translocation, triggering lysosomal cell death (LCD), while LCD promotes potassium efflux, activating NLRP3 inflammatory vesicles and pyroptosis, which leads to the production of IL-1β [29,30]. Ferroptosis is recognized as a type of autophagy-dependent cell death [31], and ferroptotic cell death can be induced after activating the STING pathway via lipid peroxidation, and this activation is independent of IFN induction [32]. cGAS-STING signaling is strongly associated with cell death, and there are signaling interactions between different cell death pathways, but the signaling dependence of different pathways differs.

#### 3.2.2. cGAS-STING Pathway and Autophagy

In addition to affecting anti-viral gene production, STING has also been demonstrated to regulate certain anti-viral cellular processes. Autophagy, a process that eliminates damaged components and prevents tumor cell outgrowth [15], can enhance antitumor effects in a STING-dependent manner by interfering with components in the autophagy pathway [33]. Additionally, the cGAS-STING pathway is regulated by autophagy, which facilitates the retrograde trafficking of STING to the ER for eventual lysosomal degradation [15]. Consequently, cells deficient in autophagy proteins or treated with drugs that inhibit lysosomal function can enhance IFN-I production [33]. While canonical autophagy is considered independent of the ULK complex and TBK1, cGAS-STING pathway-related autophagy is considered noncanonical autophagy. Following the binding of cGAMP to activate STING and its transfer to the ER-Golgi intermediate compartment (ERGIC) and the Golgi, this complex can serve as a membrane to facilitate LC3 lipidation [34]. This cGAMP-induced autophagy is important for the clearance of viruses and DNA. Correspondingly, the lack of mitophagy can initiate an immune response through the mtDNA-cGAS-STING cascade, disrupting mitochondrial homeostasis [35]. At the beginning of investigating the role of this pathway, its functions can be best characterized during *Mycobacterium tuberculosis* infection [36,37]. However, other species such as mice lack some autophagy components [38], and many pathogens have developed mechanisms to counteract autophagy [39]. Autophagy also prevents excessive inflammation by degrading activated STING with negative feedback regulation of STING signaling intensity. In summary, autophagy plays a key role in the downstream pathway of STING and contributes significantly to maintain intracellular homeostasis. However, the relationship between these two processes requires further elucidation.

#### 3.2.3. cGAS-STING Pathway and Metabolic Regulation

Beyond its role as a key immune response pathway, the cGAS-STING pathway also influences the efficiency and quality of immune responses by regulating the metabolic state of cells [9]. This pathway has been shown to regulate mitochondrial metabolism, lipid metabolism, glucose metabolism, and insulin resistance [40]. Dysregulated activation of this pathway has been linked to osteoporosis [41], nonalcoholic fatty liver disease (NAFLD) [42], and other conditions. While the etiology of diabetes has been extensively studied, a growing body of research indicates that chronic low-grade inflammation plays an important role in the development of this disease. Studies have demonstrated that the administration of a high-fat diet to mice activates the cGAS-STING pathway, resulting in an inflammatory response [43]. Additionally, increased expression of STING and some pro-inflammatory factors in obesity-induced pulmonary disease and obesity-induced lung inflammation can be effectively attenuated after STING inhibition [44]. Furthermore, during type 2 diabetes, reactive oxygen species (ROS) have been detected in pancreatic beta cells [45]. Mitochondria are important organelles that participate in many cellular processes, particularly cellular metabolism [46]. Thus, during type 2 diabetes, mtDNA release due to metabolic stress may induce mtDNA release into the cytoplasm and then lead to the activation of the cGAS-STING pathway, which in turn leads to the release of downstream-associated factors [9]. The prospect of targeting the cGAS-STING pathway in the treatment of diabetes is feasible and promising, though the specific modalities need to be further explored.

### 3.3. Relationship Between cGAS-STING Pathway and Other Inflammatory Pathways

The cGAS-STING pathway has been demonstrated to be associated with the TLR pathway, the NLRP3 inflammasome, and the MAPK pathway. Upon cGAS-STING pathway activation, downstream effector molecules (e.g., TBK1 and IRF3) can indirectly activate the MAPK pathway (e.g., p38 and JNK). NLRP3 inflammasome is a multi-protein complex that consists of NLRP3 protein, apoptosis-associated speck-like protein containing a CARD (ASC) protein, and caspase-1 [47]. The NLRP3 inflammasome requires two signals for activation. The first signal is the binding of LPS or other PAMPs to TLR4, which triggers the upregulation of NLRP3, pro-IL-18, and pro-IL-1β. The second signal is prompted by DAMPs. These two signals induce the oligomerization of NLRP3 into ASC spots, where the ASC protein generates active p20/p10 subunits through the recruitment of pro-caspase-1 followed by the autocatalytic cleavage of pro-caspase-1 [47]. Caspase-1 cleaves pro-IL-1β and pro-IL-18 to produce their active forms, and caspase-1 cleaves Gasdermin D (GSDMD), releasing its N-terminal structural domain (GSDMD-N). This cleavage creates a pore in the cell membrane, leading to cell swelling, rupture, and the release of inflammatory contents [48].

Research has demonstrated that manganese induces neuroinflammation through the mtDNA-cGAS-STING-NLRP3 axis, and STING promotes NLRP3 oligomerization via binding of its C-terminal domain to the pyrin domain (PYD) domain of NLRP3. Furthermore, STING activation triggers the release of calcium ions from the ER, thereby phosphorylating NLRP3 to enhance its activity [49]. Another study showed that in the herpes simplex virus type 1 (HSV-1) infection, STING facilitates the assembly of the NLRP3 inflammasome by recruiting NLRP3 to the ER vicinity through its ER localization. Furthermore, STING orchestrates the deubiquitination of NLRP3 by recruiting deubiquitinating enzymes, thereby promoting NLRP3 oligomerization and subsequent inflammasome activation. This study unveils a dual regulatory mechanism of STING in the spatiotemporal coordination and post-translational modification of NLRP3, providing novel therapeutic targets for the intervention of anti-viral immunity and inflammatory disorders [50].

## 4. The cGAS-STING Pathway in Pulmonary Disease

**Figure 2:**
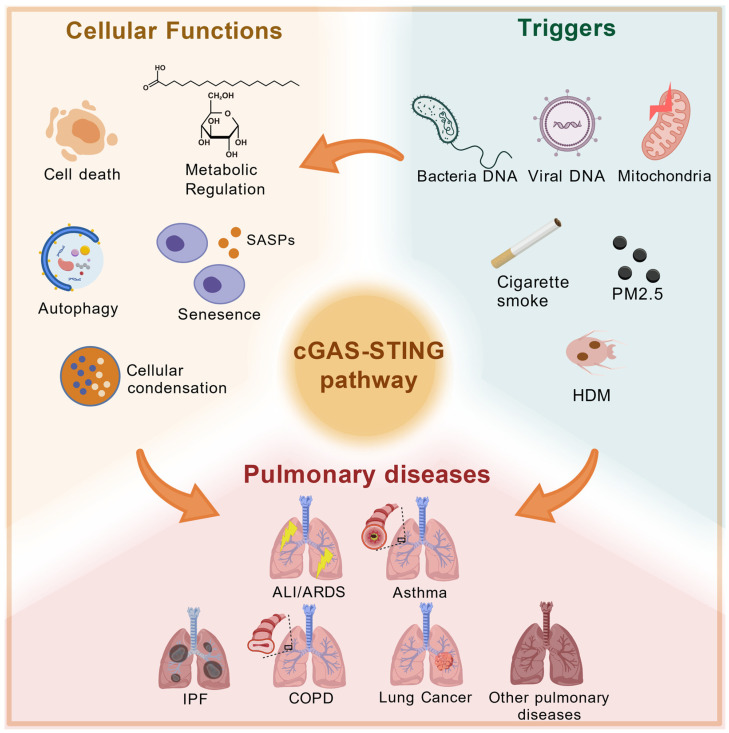
cGAS-STING pathway and pulmonary diseases. When pathogens invade the human body, the cGAS-STING pathway is activated to produce IFNs and some pro-inflammatory cytokines, triggering immune responses. Activation of this pathway can further lead to the development of various pulmonary diseases, such as ALI/ARDS, asthma, COPD, IPF, and so on.

The lung is a complex organ that terminates in a highly vascularized alveoli, which plays a role in gas exchange. Alveoli account for nearly 90 percent of the lung total volume [51]. The cGAS-STING pathway is related to the production of IFN-I, which is one of the main sources of these inflammation factors as mentioned above. Transient activation of the cGAS-STING pathway is important for the production of inflammatory factors and IFN-I to resist the invasion of external pathogens. However, sustained activation can lead to some inflammatory diseases [52]. A multitude of pulmonary diseases are linked to inflammation, such as ARDS, ALI, COPD, asthma, IPF, and so on. Furthermore, the activation of the cGAS-STING pathway contributes to the inflammatory responses observed in pulmonary diseases [53] (Figure 2).

### 4.1. Acute Respiratory Distress Syndrome (ARDS)/Acute Lung Injury (ALI)

Clinically, ALI and ARDS are pulmonary manifestations of an acute systemic inflammatory process that is characterized by diffuse pulmonary infiltrates, hypoxemia, decreased lung compliance, and edema. Hypoxic respiratory failure with bilateral pulmonary infiltrates is a syndrome associated with pulmonary and non-pulmonary risk factors [54]. The site of infection in this disease ranges from the lower respiratory tract to the lung parenchyma, leading to a shorter life expectancy. The most common causative factors include respiratory viruses, common Gram-negative or Gram-positive bacteria, sepsis, trauma, and polytrauma [55].

Li [56] et al. found that the cGAS-STING-NLRP3 axis can be activated and phosphorylated via c-Myc in LPS-induced ALI in mice. Mitochondrial dysfunction can be observed in the early stage of ALI/ARDS [57], and mtDNA levels are upregulated in BALF during ALI/ARDS [58]. LPS increases the levels of mtDNA, which further increases the activation of the cGAS-STING pathway [56]. Several studies have shown that perillaldehyde can attenuate LPS-induced ALI by inhibiting the cGAS-STING pathway, further supporting the critical role of this pathway in the pathogenesis of ALI [59]. Therefore, targeting this pathway offers a promising therapeutic strategy for the treatment of ALI.

### 4.2. Asthma

Asthma is one of the most common inflammatory lung diseases around the world and affects different age groups. It occurs in both large and small airways and is triggered by environmental factors and genomic influences [60]. The main symptoms of this disease include breathlessness, chest tightness, wheezing, and allergen sensitization [61].

Studies have shown that cytosolic dsDNA accumulates during the development of allergic inflammation induced by OVA and HDM. Genetic ablation of cGAS in mouse AECs attenuated OVA- and HDM-induced airway inflammation [24]. An analysis of nasal lavage samples from healthy and asthmatic patients following rhinovirus vaccination revealed that asthmatic patients exhibited higher symptom scores for both upper and lower respiratory tract symptoms compared to healthy individuals [62]. In addition, cGAMP exacerbated allergic inflammation in HDM-induced asthma [63]. IgE cross-linking on basophils and mast cells leads to inflammation, which prompts these cells to release chemical mediators such as leukotrienes [63], and STING plays a pivotal role in the maturation of IgE-producing B cells [64]. These observations demonstrated that the cGAS-STING pathway is involved in the pathogenesis of asthma. Targeting the cGAS-STING pathway may provide a feasible therapeutic approach for asthma.

### 4.3. Idiopathic Pulmonary Fibrosis (IPF)

IPF is a lethal, chronic inflammatory lung disease, is a constituent of the idiopathic interstitial pneumonias. It is notable for its heterogeneity in both clinical progression and fundamental biology [65]. IPF predominantly affects middle-aged and elderly populations, and currently available treatments remain ineffective. It is characterized by the presence of paraseptal fibrosis and honeycombing areas in the pulmonary tissue, resulting in impaired pulmonary function, progressive lung scarring, and collagen deposition. This combination of factors contributes to a high mortality rate [65].

According to different clinical forms, it can be divided into four stages: slowly progressive course, accelerated course, acute exacerbation of idiopathic pulmonary fibrosis (AE-IPF), and IPF with other pulmonary diseases [66]. IPF is also characterized by distinct phenotypes, including combined pulmonary fibrosis and emphysema (CPFE), pulmonary hypertension associated with IPF, and rapidly progressive IPF [67].

AECs senescence is a hallmark of the general IPF process. In this way, many studies have begun to investigate the relationship between AEC senescence and cGAS [68]. Schuliga et al. confirmed that cGAS-dependent response can augment AEC senescence, which means cGAS is a potential target for IPF [68]. cGAS activation has been observed in the fibroblast regions from the lungs of IPF patients. The knockdown of cGAS or the use of siRNAs to inhibit cGAS has been demonstrated to attenuate the progression of IPF senescence [2]. Particulate matter 2.5 (PM2.5) is a common inducer in aging-related lung diseases. Wang et al. used PM2.5 in A549 cells and found that the increase in cell senescence depended on the PM2.5 concentration. Using cGAS inhibitors can alleviate this process, which is related to the cGAS-STING-NF-κB pathway [69].

AE-IPF is a major cause of death in IPF patients [70]. TMEM173 encodes the protein STING, and TMEM173 mutations have been identified in AE-IPF patients, suggesting that STING might be involved in the progression of AE-IPF [71]. Sun et al. [72] showed that Juglanin treatment alleviates bleomycin-triggered lung fibrosis by inhibiting the STING signaling pathway in mice, which indicate that STING is involved in the pathogenesis of bleomycin-induced IPF. However, other studies have found that STING plays a totally opposite role in IPF. Savigny et al. found that STING deficiency will lead to the exacerbation of pulmonary fibrosis; STING plays a protective role in BLM-induced lung fibrosis, and STING’s protective role may rely on neutrophilic inflammation resolution [73]. In conclusion, targeting the cGAS-STING pathway has great potential in the treatment of IPF.

### 4.4. Chronic Obstructive Pulmonary Disease (COPD)

COPD encompasses a group of diseases in which the inhalation of a toxic substance triggers a chronic and inflammatory response in the airways, resulting in airflow limitation [74]. This chronic disease is the third leading cause of death around the world [75]. The etiology of COPD is multifactorial, with cigarette smoke being the most common inducer, followed by other pollutants [74]. The inflammatory response in COPD is driven by DNA damage and cell death, which are induced by excessive ROS production [76]. As COPD progresses, DAMPs are significantly increased and recognized by TLRs, which subsequently activate NF-κB [77]. Furthermore, studies have identified that autoimmune elements participate in COPD pathology, as the release of self-DNA from dying lung cells can trigger an immune response via cGAS activation [78]. Moreover, elevated cell-free DNA levels have been associated with an increased risk of adverse outcomes in COPD patients. NETs are a web-like structure of self-DNA released by neutrophils and one of the main mechanisms in the pathogenesis of COPD [79]. In clinical patients, the formation of NETs is elevated in both stable and exacerbated COPD [80]. Chen et al. demonstrated that targeting NETs and the cGAS-TLR9 axis can alleviate COPD-related airway inflammation [77]. In addition, COPD frequently involves dsDNA, which is activated by DNA sensing pathways. STING is highly expressed in COPD fibroblasts compared with healthy fibroblasts. Mdkhana [81] et al. demonstrated that the STING pathway is involved in the COPD pathogenesis, which indicates that the combination of STING inhibitor and steroid could be a viable treatment option. In cigarette smoking exposure-induced COPD, self-DNA is released and sensed by the cGAS-STING pathway, leading to IFN-I secretion [82]. In summary, the above evidence clearly establishes the significance of the cGAS-STING signaling pathway in the development and potential treatment of COPD.

### 4.5. Lung Cancer

Lung cancer remains a deadly global health problem, and the overall survival rate is nearly 18 percent at five years [83]. The cGAS-STING pathway exerts anti-tumor effects through two different mechanisms: autonomous and non-autonomous [84]. The production of downstream cytokines contributes to the promotion of dendritic cell (DC) cross-presentation and the initiation of tumor-specific CD8+T cell responses [84]. Immunotherapy constitutes a significant treatment modality, such as immune checkpoint inhibitor (ICI) and adoptive cell transfer therapy. However, some patients exhibit an ineffective response to these treatments [85].

Recent studies have indicated that cGAS plays a pivotal role in the effectiveness of immune checkpoint inhibition therapy. It has been shown to induce anti-tumor immune responses and enhance the effects of immune checkpoint inhibitors by activating the STING pathway. In the absence of cGAS or when its function is impaired, the efficacy of immune checkpoint inhibition therapy is significantly diminished, suggesting that the presence of cGAS is a crucial factor in the success of tumor immunotherapy [86]. The tumor microenvironment is a complex system. Recent studies have demonstrated that a robust immune response can be triggered within the tumor microenvironment through the direct activation of the STING signaling pathway. This activation has been shown to directly destroy tumor cells and induce a systemic immune response, enabling the immune system to recognize and eliminate distant tumor cells [87].

The cGAS-STING pathway plays dual roles in lung cancer. One is pro-tumorigenic, mediated by inflammatory responses, while the other promotes anti-tumor responses by enhancing the capacity for cross-presentation of DCs. This enables DCs to present tumor antigens more effectively to T cells [85]. Furthermore, cancer-induced DNA damage mediates cellular senescence through the cGAS-STING pathway, which promotes cellular senescence. This process is largely dependent on the expression of a number of cytokines and growth factors, which consist of senescence-associated secretory phenotype (SASP) components [84]. Lung cancer cells develop multiple intrinsic mechanisms to inhibit the cGAS-STING pathway, avoiding surveillance and attack by the immune system by inhibiting this pathway. Therefore, the cGAS-STING pathway is a key target in the immune escape mechanism of lung cancer [88]. Consequently, the activation of the cGAS-STING pathway is considered as a promising immunotherapeutic strategy.

Non-small cell lung cancer (NSCLC) takes up nearly 85 percent of lung cancer patients, and radiotherapy plays an instrumental role in its treatment [89]. STING is widely expressed in NSCLC, and its expression is associated with T cell functions. Loss of STING leads to poor prognosis [90]. Radiotherapy produces DNA double-strand breaks (DSBs), which can activate the cGAS-STING pathway via a DNA-sensing mechanism. Xue et al. found that activating the cGAS-STING pathway could promote the radiosensitivity of NSCLC cells, suggesting that targeting the cGAS-STING pathway could be a strategy for NSCLC radiosensitization [91]. Another study aimed to determine the role of cGAS in promoting NSCLC cell proliferation. Liu et al. showed that arginine methylation modulates cGAS stability and that the protein arginine methyltransferase 1 (PRMT1)-cGAS-USP7 axis is a therapeutic target for NSCLC. Additionally, the study revealed a correlation between the expression of cGAS and PRMT1 in human NSCLC [92].

ICI treatment is a common therapeutic approach in cancer, which mainly includes anti-programmed death-1/programmed death-ligand 1 (PD-1/PD-L1) and anti-cytotoxic T-lymphocyte-associated protein 4 (CTLA-4) [93]. STING agonists increase the amounts of T cell infiltration into tumor, which helps relieve resistance to anti-PD-1/PD-L1 [94]. Furthermore, Harding et al. demonstrated that the cGAS-STING pathway enhances the effect of anti-CTLA-4 treatment [95]. Therefore, combining STING agonists with ICI treatment has great therapeutic potential.

### 4.6. Coronavirus Disease 2019 (COVID-19)

Coronavirus is an RNA virus. Severe acute respiratory syndrome coronavirus 2 (SARS-CoV-2), discovered in 2019, is a member of the Coronaviridae family. The spike (S) protein is a crucial structural protein located on the surface of SARS-CoV-2 and is a key to the virus’s entry into host cells. The S protein binds to the host cell surface receptor ACE2, facilitating viral entry and the release of viral RNA into the host cell, thereby initiating infection [96].

The immune sensing pathways are activated through dsRNA and viral single-stranded RNA (ssRNA) combining with PRRs via RIG-I like receptors (RLRs) and TLRs to initiate these pathways [97]. The initial phase of COVID-19 infection is marked by the increased permeability of the mitochondrial membrane, which results in the leakage of mitochondrial DNA (mtDNA) into the cytoplasm. cGAS activates the STING signaling pathway by recognizing free DNA released from SARS-CoV-2, including viral replication intermediates or mtDNA released due to mitochondrial stress. This activation triggers the production of IFN-I and pro-inflammatory cytokines, forming a crucial component of the innate immune defense [98]. In COVID-19, the STING-TBK1-IRF3 axis can be blocked by the virus protease [97]. In addition, the levels of IFN-I represent the severity of COVID-19, and low IFN-I levels are associated with severe disease progression [99]. Nevertheless, the virus has evolved sophisticated immune evasion strategies. Primarily, it can directly suppress STING functionality through viral proteins such as ORF3a and nonstructural protein 6 (NSP6) (inhibits IFN production and promotes autophagy-mediated STING degradation) [100]. Additionally, molecular interference with signal transduction is achieved via viral components, including papain-like proteases (suppresses the anti-viral pathway through the process of deubiquitinating the stimulator of STING) and nucleocapsid protein [101]. Currently, two main research strategies are employed. The first is to adopt different treatment approaches at different stages of the disease. In the early stages, cGAS-STING agonists are used to activate the signaling pathway, thereby enhancing the anti-viral response. Conversely, in the later stages, corresponding inhibitors are employed to suppress STING and reduce tissue damage [98]. The second treatment strategy involves targeted therapy, such as using STING agonists to activate STING and enhance the secretion of IFN-I, or using combination therapy with anti-viral drugs and STING modulators [102].

### 4.7. Other Pulmonary Diseases

The cGAS-STING pathway is implicated in a variety of pulmonary diseases. Pulmonary arterial hypertension (PAH) is a fatal vascular disorder characterized by lung vascular remodeling and increased vascular resistance [103]. A study shows that calcitonin gene-related peptide (CGRP) attenuates vascular remodeling via the cGAS-STING-NF-κB pathway in PAH [104]. Therefore, the inhibition of this pathway may provide a promising therapeutic strategy. Li et al. found that β-sitosterol (SITO) is a useful agent for PAH vascular remodeling as it inhibits PASMC proliferation via the DNA damage/cGAS-STING pathway [105]. Tuberculosis is a fatal communicable disease caused by *M. tuberculosis* and leads to substantial chronic lung disability [106]. Collins et al. demonstrated that cGAS is a pivotal innate sensor of *M. tuberculosis* infection and that both STING and cGAS are required for *M. tuberculosis*-induced selective autophagy [107]. During the infection process, mitochondria and *M. tuberculosis* release dsDNA to activate the cGAS-STING pathway, subsequently leading to the release of IFN-I. This cytokine enhances antimicrobial responses by modulating the function of immune cells (e.g., macrophages and T cells) and contributes to the clearance of *M. tuberculosis* [108]. Therefore, investigating the cGAS-STING pathway can provide a novel therapeutic approach for tuberculosis. Silica exposure is a common inducer of lung inflammation, Benmerzoug [109] et al. found that exposure to silica dust induces sterile lung inflammation, which in turn promotes a type 2 immune response through the STING signaling pathway, thereby exacerbating the progression of *M. tuberculosis* infection.

As described above, the cGAS-STING signaling pathway plays a dual role in lung homeostasis and pathology. In the context of lung cancer, COVID-19, and other virus-induced lung injuries, activation of the cGAS-STING pathway exerts a protective effect. When a virus invades the host, its dsDNA is recognized by cGAS, which in turn activates STING. This transient activation is crucial as it promotes the production of inflammatory factors and IFN-I, which is pivotal to activate immune cells to combat invading pathogens. However, when the cGAS-STING signaling pathway is persistently or inappropriately activated, it can contribute to the pathogenesis of chronic inflammatory diseases, such as IPF and COPD. Cell death-induced release of self-DNA activates the cGAS-STING pathway, driving the production of IFN-I. Under these conditions, inhibiting the cGAS-STING pathway confers a protective effect. In conclusion, the role of this pathway in different lung diseases largely depends on the disease context, the source of activation signals (exogenous pathogen DNA or endogenous self-DNA), the intensity of activation (acute or chronic), and the overall immune status of the host. Thus, understanding these underlying mechanisms is crucial for the development of therapeutic strategies targeting the cGAS-STING pathway.

## 5. Agonists and Inhibitors of cGAS-STING Pathway

### 5.1. Agonists

The primary activation mechanism involves a shift from the autoinhibition form of STING, resulting in the disability of bilayers and transfer to a curved monolayer activated state. Agonists of the cGAS-STING pathway can be categorized into two distinct groups: one group targets the cytosolic cyclic di-nucleotide binding domain (CBD), while another targets the transmembrane domain (TMD) [110]. Agonists of the cGAS-STING pathway can be broadly categorized into natural agonists and synthetic agonists. Natural agonists primarily include pathogen-derived DNA (e.g., bacterial or viral DNA), mtDNA, and nuclear DNA released during periods of cellular stress or damage. Synthetic agonists encompass dsDNA analogs (e.g., ISD or poly (dA:dT)), cGAMP analogs (e.g., 2′3′-cGAMP), and small-molecule STING agonists, such as mouse-specific agonists (e.g., DMXAA), human-specific agonists (e.g., ADU-S100/MIW815), and non-nucleotide oral agonists (e.g., diABZI).

STING agonists commonly focus on tumor immunotherapy and vaccine adjuvants. Especially in tumor immunotherapy, the combination with photothermal, photodynamic, and sonodynamic therapies represents an effective anti-tumor strategy [111]. Currently, many STING agonists are undergoing clinical trials. DMXAA is the most widely used and well-characterized compound among STING agonists. Due to its promising properties, it has been tested in several Phase I clinical trials. However, most of these trials were terminated due to poor efficacy [112]. Structural studies have revealed that DMXAA specifically binds to the STING protein in mice but not in humans, which explains the lack of therapeutic efficacy [113]. Currently, several other STING agonists are undergoing Phase I/II clinical trials, but none have yet advanced to Phase III due to side effects [112]. This highlights the need for further exploration of STING agonists in clinical applications and for the design of drugs more effectively targeting human STING. Some new STING agonists, such as BMS-986301 and BI1387446, are currently undergoing clinical trials either as monotherapies or in combination with other anti-cancer therapies [114]. Developing new STING delivery platforms is a new therapy direction, such as using nanoparticles, liposome, and polymers to load STING agonists and so on [115].

### 5.2. Inhibitors

The development of specific cGAS inhibitors remains in its early stage, with strategies classified into two categories: competitive inhibition at the catalytic site and interference with DNA binding. The former uses structural mimics of endogenous cGAS substrates to competitively occupy the substrate-binding pocket, suppressing enzymatic activity. The latter strategy involves competitive DNA binding, disrupting cGAS-DNA complex formation by occupying the DNA interaction interface and hindering cGAS activation [110]. RU.521 is shown to occupy the catalytic site of m-cGAS to block its activation [116]. G108 binds to the apo form of the h-cGAS apo catalytic domain (designated h-cGASCD) and occupies the ATP-binding site in the catalytic pocket to inhibit cGAS activation. G150 has also been shown to occupy the ATP/GTP-binding pocket [117]. An et al. found that hydroxychloroquine (HCQ) acts by binding to the DNA minor groove when cGAS binds to DNA, thereby competitively inhibiting the DNA-cGAS interaction [118]. The small molecule (PF-06928215) can competitively occupy the DNA binding pocket [119], and oligonucleotides (A151) block DNA binding by spatial site-blocking [120] (Table 1). Studies have found that interfering with SPSB3, a cGAS substrate receptor, to regulate the degradation of nuclear cGAS can activate the IFN-1 signaling pathway, providing new insights and potential therapeutic avenues for combating viral infections [121]. To date, three cGAS inhibitors have entered Phase I clinical trials. Among them, VENT-03 is the first oral cGAS inhibitor in the world to successfully complete its first-in-human Phase I trial, demonstrating good targeted efficacy, and there are plans to initiate Phase II clinical trials [122].

**Figure 3:**
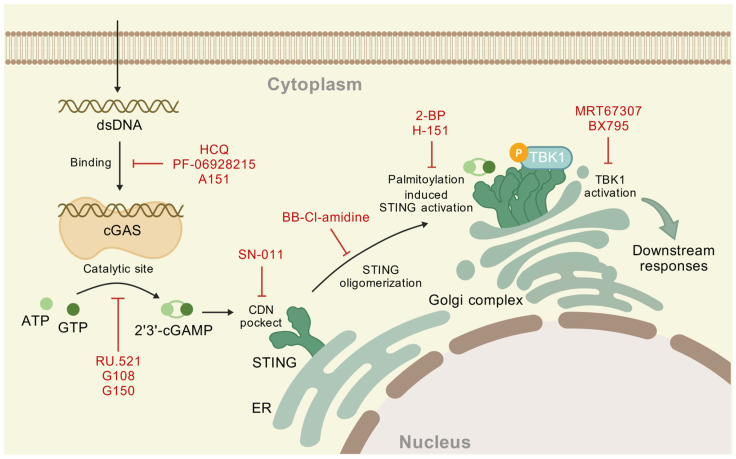
Inhibitors of the cGAS-STING pathway. These inhibitors are primarily divided into two categories: cGAS inhibitors and STING inhibitors. cGAS inhibitors include DNA binding inhibitors and catalytic site inhibitors. STING inhibitors function through four main mechanisms: interference with the CDNs-binding pocket, suppression of STING oligomerization, inhibition of palmitoylation, and inhibition of TBK1 activation. By targeting key proteins within this pathway, these inhibitors offer promising therapeutic alternatives for the treatment of pulmonary diseases.

STING homeostasis is regulated by protein–protein interactions and molecular modifications, including phosphorylation, oxidation, polyubiquitination, and carbonylation [123]. STING inhibitors can be categorized into three groups: palmitoylation inhibitors, TBK1 inhibitors, and cyclic dinucleotide (CDN) pocket inhibitors [124]. Among these groups, preventing STING palmitoylation represents a particularly effective method. 2-bromopalmitate (2-BP), a de-palmitoylation compound, has been shown to reduce the expression of downstream cytokine genes by inhibiting STING palmitoylation [123]. In addition, the mutation of Cys residues (Cys88/91) has been observed to suppress palmitoylation. H151 can block the palmitoylation of STING by modifying Cys91 [125]. H151 has been demonstrated to play a pivotal role in the treatment of inflammatory lung injury [126]. MRT67307 acts as a TBK1 inhibitor, which inhibits IRF3 phosphorylation and thereby influences the cGAS-STING pathway function [127]. BX795, another inhibitor of TBK1 and IKKε, contributes to the competitive inhibition of ATP binding [128]. Recent studies have demonstrated that SN-011 has a stronger affinity for the CDNs pocket than 2′3′-cGAMP. It stabilizes STING in an open, inactive conformation, thereby suppressing the 2′3′-cGAMP-induced production of interferons and inflammatory cytokines [129]. In addition to these inhibition modes, BB-Cl-amidine inhibits STING oligomerization by modifying Cys148 [130] and then inhibits the activation of downstream pathways (Table 2) (Figure 3).

STING, located at the center of the signaling pathway, is not only regulated by upstream factors but also influences the phosphorylation of downstream proteins, such as TBK1 and IRF3. Therefore, targeted inhibition of STING has become the focus of current research. Recent studies have elucidated the mechanism of negative regulation of STING, where adaptor protein complex 1 (AP-1) sorts phosphorylated STING into clathrin-coated transport vesicles, which are then delivered to the endolysosomal system for degradation. This finding provides a foundational basis for the development of novel STING inhibitors [131]. Compared with STING agonists, the development of STING inhibitors is still in its early stages, and no candidate drugs have entered clinical trials yet [132]. The STING inhibitors reported so far mostly suffer from issues such as insufficient activity, species sensitivity, and poor pharmacokinetic properties, which limit their clinical application.

The research and development of these inhibitors provides some new directions and therapeutic strategies for the future studies of the cGAS-STING pathway.

## 6. Perspectives

The mechanistic underpinnings of the cGAS-STING pathway have been extensively characterized, with accumulating evidence elucidating its critical pathophysiological relevance in pulmonary disorders. Pharmacological modulation of the cGAS-STING pathway through targeted agonism or antagonism demonstrates significant potential for ameliorating disease progression, thereby paving the way for novel therapeutic modalities in respiratory medicine. In this review, we summarize the role of the cGAS-STING signaling pathway in different lung diseases, highlighting its pivotal role in treatment. However, further research is needed to accelerate its clinical implementation.

Future research should focus on further exploring the interactions of this pathway with other pathways involved in different lung diseases, as well as the potential for developing targeted drug development and evaluating their clinical outcomes in trials. The combination of cGAS-STING targeted therapy with other therapeutic modalities has been demonstrated to have significant therapeutic potential. Therefore, exploring more combination therapies is necessary. For example, the combination of cGAS-STING pathway agonists and ICIs has demonstrated significant potential in the treatment of lung cancer. The STING signaling pathway has been demonstrated to enhance tumor immune surveillance by promoting tumor T cell infiltration [133] and increasing the expression of antigen-presenting molecules [134]. As previously mentioned, the cGAS-STING pathway plays a dual role in the pathogenesis of IPF; this phenomenon highlights the pathological complexity and microenvironment-dependent nature of different lung diseases, representing a critical direction for future research, such as diverse cellular functions and the impacts of specific modes of activation.

Further exploration is needed into the relationship between the cGAS-STING pathway and specific rare pulmonary diseases. This includes certain airway disease (e.g., bronchiectasis) and non-IPF fibrotic diseases (e.g., radiation pneumonitis). Technologies like single-cell sequencing and spatial transcriptomics can also be employed to resolve the spatiotemporal heterogeneity of cGAS-STING signaling in various cell types at different stages of lung diseases.

## 7. Conclusions

In summary, research on the cGAS-STING pathway in pulmonary diseases still requires further refinement and exploration, and this pathway holds great potential for the treatment of pulmonary diseases. It is believed that in the future, by further elucidating the mechanisms underlying the involvement of different cell types in pulmonary diseases and the crosstalk between the cGAS-STING signaling pathway and other signaling pathways, more research on combination therapies will provide stronger foundation for developing novel treatments for lung diseases. Furthermore, the application of genomics and transcriptomics may facilitate a more profound comprehension of this pathway. And to advance drug development, this target requires further validation of its efficacy through precise preclinical in vivo and early-stage clinical trials.

## Figures and Tables

**Table 1:** Inhibitors of cGAS.

Function	Compound	Potential Mechanism	Reference
Disrupt DNA binding	hydroxychloroquine (HCQ)	binds to DNA minor groove	[118]
	PF-06928215	binds to the cGAS active site	[119]
	A151	competitive with DNA	[120]
Inhibit catalytic site	RU.521	occupies the catalytic site of m-cGAS	[116]
	G108	occupies ATP binding location	[117]
	G150	occupies the catalytic site of h-cGAS	[117]

**Table 2:** Inhibitors of STING.

Function	Compound	Potential Mechanism	Reference
CDNs pocket inhibitors	SN-011	compete for the STING binding pocket	[129]
STING oligomerization inhibitors	BB-Cl-amidine	modify Cys148	[130]
Palmitoylation inhibitors	2-BP	blocking S-palmitoylation of STING	[123]
	H-151	modifying Cys91 to block palmitoylation	[125]
TBK1 inhibitors	MRT67307	dual inhibitor of TBK1/IKKε inhibits IRF3 and NF-κB pathways by blocking kinase activity	[127]
	BX795	blocking of STING-TBK1 signal	[128]

## Data Availability

No new data were created or analyzed in this study. Data sharing is not applicable to this article.
